# Performance of *MYC*, *BCL2*, and *BCL6* break-apart FISH in small biopsies with large B-cell lymphoma: a retrospective Cytopathology Hematopathology Interinstitutional Consortium study

**DOI:** 10.3389/fonc.2024.1408238

**Published:** 2024-06-06

**Authors:** Joshua R. Menke, Umut Aypar, Charles D. Bangs, Stephen L. Cook, Srishti Gupta, Robert P. Hasserjian, Christina S. Kong, Oscar Lin, Steven R. Long, Amy Ly, Jacob A. S. Menke, Yasodha Natkunam, Roberto Ruiz-Cordero, Elizabeth Spiteri, Julia Ye, Sara L. Zadeh, Dita A. Gratzinger

**Affiliations:** ^1^ Division of Hematopathology, Department of Pathology, Stanford University, Stanford, CA, United States; ^2^ Division of Cytogenetics, Department of Pathology and Laboratory Medicine, Memorial Sloan Kettering Cancer Center, New York, NY, United States; ^3^ Division of Cytogenetics, Department of Pathology, Stanford University, Stanford, CA, United States; ^4^ Department of Laboratory Medicine, San Francisco Veterans Administration Health Care System, San Francisco, CA, United States; ^5^ Division of Hematopathology, Department of Laboratory Medicine, San Francisco, CA, United States; ^6^ Division of Hematopathology, Department of Pathology, Massachusetts General Hospital, Boston, MA, United States; ^7^ Division of Cytopathology, Department of Pathology, Stanford University, Stanford, CA, United States; ^8^ Division of Cytopathology, Department of Pathology and Laboratory Medicine, Memorial Sloan Kettering Cancer Center, New York, NY, United States; ^9^ Division of Cytopathology, Department of Pathology, University of California, San Francisco, San Francisco, CA, United States; ^10^ Division of Cytopathology, Department of Pathology, Massachusetts General Hospital, Boston, MA, United States; ^11^ Senior Backend Engineer, Big Nerd Ranch, Atlanta, GA, United States; ^12^ Divisons of Molecular Genetic Pathology, Cytopathology, and Hematopathology, Department of Pathology and Laboratory Medicine, University of Miami, Miami, FL, United States; ^13^ Division of Cytopathology, Department of Pathology, University of Virginia, Charlottesville, VA, United States

**Keywords:** diffuse large B-cell lymphoma, high-grade B-cell lymphoma, double-hit lymphoma, FISH, BCL2 rearrangement, MYC rearrangement

## Abstract

**Introduction:**

Fluorescence *in situ* hybridization (FISH) is an essential ancillary study used to identify clinically aggressive subsets of large B-cell lymphomas that have *MYC, BCL2*, or *BCL6* rearrangements. Small-volume biopsies such as fine needle aspiration biopsy (FNAB) and core needle biopsy (CNB) are increasingly used to diagnose lymphoma and obtain material for ancillary studies such as FISH. However, the performance of FISH in small biopsies has not been thoroughly evaluated or compared to surgical biopsies.

**Methods:**

We describe the results of *MYC, BCL2,* and *BCL6* FISH in a series of 222 biopsy specimens, including FNAB with cell blocks, CNBs, and surgical excisional or incisional biopsies from 208 unique patients aggregated from 6 academic medical centers. A subset of patients had FNAB followed by a surgical biopsy (either CNB or excisional biopsy) obtained from the same or contiguous anatomic site as part of the same clinical workup; FISH results were compared for these paired specimens.

**Results:**

FISH had a low hybridization failure rate of around 1% across all specimen types. FISH identified concurrent *MYC* and *BCL2* rearrangements in 20 of 197 (10%) specimens and concurrent *MYC* and *BCL6* rearrangements in 3 of 182 (1.6%) specimens. The paired FNAB and surgical biopsy specimens did not show any discrepancies for *MYC* or *BCL2* FISH; of the 17 patients with 34 paired cytology and surgical specimens, only 2 of the 49 FISH probes compared (4% of all comparisons) showed any discrepancy and both were at the *BCL6* locus. One discrepancy was due to necrosis of the CNB specimen causing a false negative *BCL6* FISH result when compared to the FNAB cell block that demonstrated a *BCL6* rearrangement.

**Discussion:**

FISH showed a similar hybridization failure rate in all biopsy types. Ultimately, *MYC, BCL2*, or *BCL6* FISH showed 96% concordance when compared across paired cytology and surgical specimens, suggesting FNAB with cell block is equivalent to other biopsy alternatives for evaluation of DLBCL or HGBCL FISH testing.

## 1 Introduction

**Figure 1:**
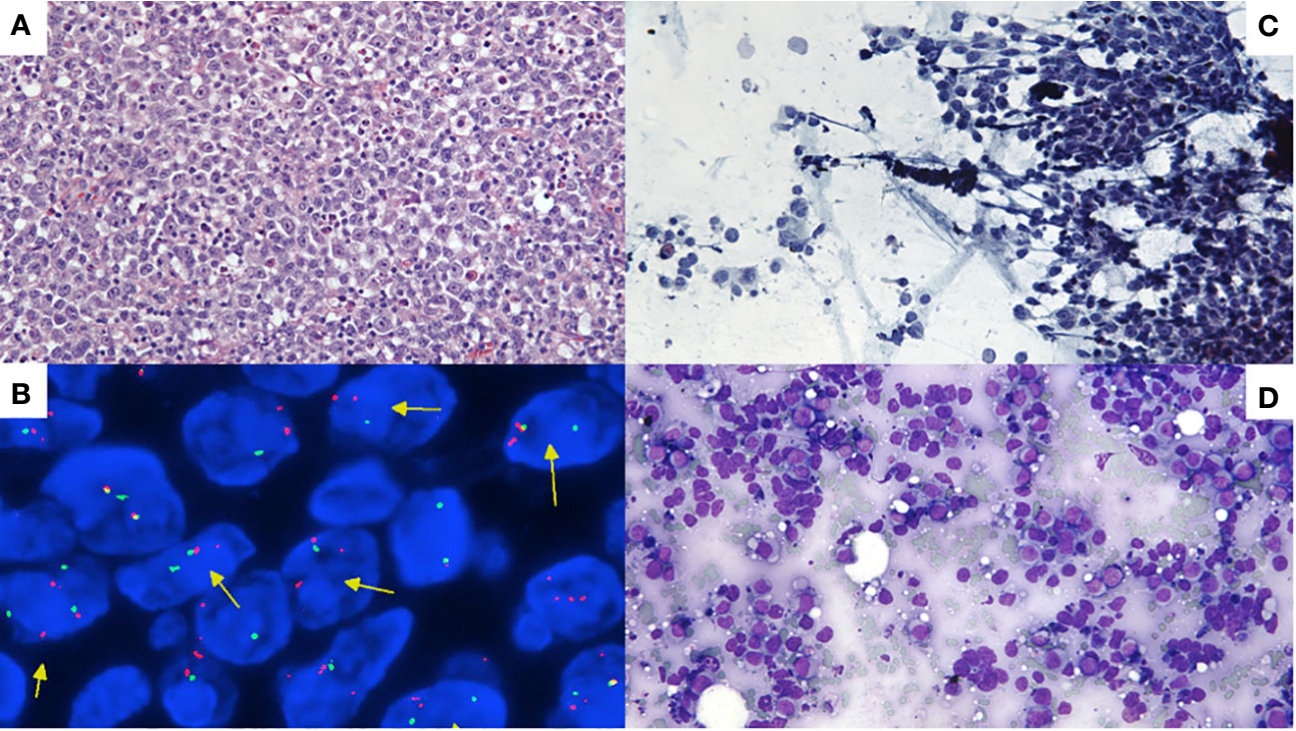
**(A)** H&E-stained section of lymph node cell block demonstrating effacement by high-grade B-cell lymphoma with *MYC* and *BCL2* rearrangements. **(B)** *MYC* interphase FISH showing separation of orange and green signals indicating a *MYC* rearrangement (see arrows). **(C)** Pap-stained smear slide showing aggregates of large atypical lymphoid cells. **(D)** May-Grünwald Giemsa (MGG)-stained smear slide of cytology smear slide similarly showing large cells. In general, Pap stains and MGG stains are complementary and helpful to obtain in all lymph node FNAB: Pap-stained slides demonstrate greater nuclear detail but make cells appear smaller and have poor cytoplasmic detail while May-Grünwald Giemsa stains show greater cytoplasmic detail and larger cell size but poor nuclear detail.

An important subset of diffuse large B-cell lymphoma (DLBCL) and high-grade B-cell lymphoma (HGBCL) have *MYC* and *BCL2* rearrangements Large B-cell lymphoma or high-grade B-cell lymphoma with *MYC* and *BCL2* rearrangements are commonly referred to as "double-hit" lymphoma (DHL), which portends more aggressive clinical behavior and inferior progression-free survival compared to other cases of diffuse large B-cell lymphoma, germinal center B-cell subtype, or other high-grade B-cell lymphoma (1–5). Both the 5^th^ Edition of the World Health Organization Classification of Haematolymphoid tumors (WHO5) (6) and the International Consensus Classification of Lymphoid Neoplasms (ICC) (7) now classify diffuse large B-cell lymphoma and high-grade B-cell lymphoma with concurrent *MYC* and *BCL6* “double-hit” lymphomas separately due to the unclear prognostic significance of this combination, with some studies not showing distinct biology for these cases (1, 2), but other studies demonstrating an association with a poor outcome (3, 8–11). Fluorescence *in situ* hybridization (FISH) is a frequently used technique to detect *MYC*, *BCL2*, and *BCL6* rearrangements. Because diffuse large B-cell lymphoma and other high-grade B-cell lymphoma can look identical morphologically to “double-hit” lymphoma, at a minimum *MYC* FISH must be obtained in every case (see **Figure 1** for an example of a case that has similar morphology to diffuse large B-cell lymphoma, NOS on various slide stain preparations, but ended up having both *MYC* and *BCL2* rearrangements). In one survey of cytogenetics laboratories, the most common test strategy (67%) was upfront testing of *MYC*, *BCL2*, and *BCL6* FISH on every case while fewer labs (26%) performed *BCL2* and *BCL6* FISH testing only if *MYC* is rearranged (12). In the same survey study, 56% of laboratories performed *MYC* break-apart probe (BAP) in combination with *IGH/MYC* dual fusion probe while 43% of laboratories performed only *MYC* BAP. MYC and BCL2 IHC are known to be poor predictors of *MYC* rearrangements at the gene level and polymorphisms have even been shown to create false negative MYC IHC results (13). Data are less clear regarding *MYC* and *BCL6* rearrangements, but many institutions continue to test for *BCL6* rearrangements given some data indicating these cases may have an inferior outcome (3, 8–11).

Fine needle aspiration biopsy (FNAB) is increasingly used for triage of lymphadenopathy and diagnosis of lymphoma. Additionally, cell blocks (CB) and smear slides from FNAB can effectively be used for FISH in various neoplasms (14–17). However, only a few, small single institutional studies have described FISH (such as *MYC*) in FNAB smears and cell block for lymphoma (18–21) and no comparisons to core biopsy or the gold standard excisional biopsy exist to our knowledge. We have two principal aims in this multi-institutional study of FISH performance in diffuse large B-cell lymphoma and high-grade B-cell lymphoma: 1) compare the success rate of *MYC, BCL2*, and *BCL6* FISH hybridization across FNAB with cell block, core biopsy, and excisional biopsy, 2) compare FNAB to either core biopsy or excisional biopsy for FISH from the same patient.

## 2 Materials and methods

Six academic medical centers participated in this study. The medical centers were assigned data access groups in REDCap (see section below on REDCap) and they consisted of Massachusetts General Hospital (MGH), Memorial Sloan Kettering Cancer Center (MSKCC), San Francisco Veterans Administration Health Care System (SFVAHCS), Stanford, University of California San Francisco (UCSF), and University of Virginia (UVA).

A retrospective search was conducted of the pathology informatics systems with keywords “FISH” AND “diffuse large B-cell lymphoma” OR “high-grade B-cell lymphoma” over the 10-year period from 1/1/2010 to 12/31/2019. A complementary search was performed of cytogenetics lab data for all *MYC* FISH studies performed on cytology samples to catch cases missed by the pathology data system and then identify paired surgical samples through the pathology archive. Only specimens that had *MYC* FISH performed were included (n=222); of these cases, 200 had *BCL2* FISH performed (90%) and 186 (84%) *BCL6* FISH performed. Exclusion criteria included bone marrow biopsy specimens (due to alternative fixation used in some of these specimens and decalcification that could cause false negatives), and body fluid specimens (due to the numerous pre-analytic variables such as fixative type that might influence FISH performance in cell blocks). The age of the patient was recorded for each biopsy, but for **Tables 1**, **2**, the age of the patient was determined as the age at the first biopsy specimen. The WHO4R classification terminology “high-grade B-cell lymphoma with *MYC* and *BCL2* and/or *BCL6* rearrangements” was originally used for “double hit” or “triple hit” cases in this patient biopsy cohort with an endpoint in 2019 irrespective of whether the morphology was more in keeping with diffuse large B-cell lymphoma or high-grade B-cell lymphoma (22). In keeping with the WHO5 and ICC, we subsequently distinguished *MYC* and *BCL2* rearranged cases from *MYC* and *BCL6* rearranged cases. This manuscript uses the terms high-grade B-cell lymphoma-*MYC/BCL2* or high-grade B-cell lymphoma-*MYC/BCL6* to distinguish these two groups regardless of whether the morphology was originally interpreted as diffuse large B-cell lymphoma or high-grade B-cell lymphoma.

**Table 1:** Clinical characteristics of all 208 patients included in study.

Number of patients	208
Age (mean (SD))	63.65 (16.55)
Gender = Male (%)	123 (59.1)
Gender = Female (%)	83 (40.3)
Data Access Group (%)	
MGH	25 (12.0)
MSKCC	13 (6.2)
SFVAHCS	13 (6.2)
Stanford	134 (64.4)
UCSF	21 (10.1)
UVA	2 (1.0)
History of B-cell non Hodgkin lymphoma? = Yes (%)	81 (39.3)
Prior large B cell lymphoma diagnosis (%)	23 (29.1)
DLBCL, NOS	22 (95.6)
Prior follicular lymphoma diagnosis (%)	52 (64.2)
Classic follicular lymphoma (grade 1–2)	42 (80.8)
Classic follicular lymphoma (grade 3A)	6 (11.5)
other or unknown	2 (3.8)
Primary cutaneous follicle center lymphoma	2 (3.8)
Prior diagnosis of low grade B cell lymphoma (%)	13 (16.5)
CLL/SLL	4 (30.8)
EMZL	2 (15.4)
LPL	2 (15.4)
Mantle cell lymphoma	1 (7.7)
NMZL	1 (7.7)
other or unknown	1 (7.7)
Primary cutaneous MZL	1 (7.7)
SMZL	1 (7.7)
High-grade B-cell lymphoma diagnosis (%)	4 (5.1)
Burkitt lymphoma	1 (25.0)
High grade B-cell lymphoma, with MYC and BCL2 rearrangements	1 (25.0)
High grade B-cell lymphoma, NOS	2 (50.0)
Prior solid organ or stem cell transplant (%)	16 (7.7)
Stem cell transplant	9 (56.2)
Solid organ transplant	7 (43.8)
Prior chemotherapy (%)	52 (26.0)
Reason for chemotherapy (%)	
carcinoma	4 (7.7)
carcinoma and lymphoma	1 (1.9)
lymphoma	46 (88.5)
other or unknown	1 (1.9)

The classification used for all diagnoses was the WHOR4, which is equivalent to WHO5 and ICC for these diagnoses. Abbreviations used: CLL/SLL (chronic lymphocytic leukemia/small lymphocytic lymphoma), EMZL (extranodal marginal zone lymphoma), LPL (lymphoplasmacytic lymphoma), NMZL (nodal marginal zone lymphoma), MZL (marginal zone lymphoma), SMZL (splenic marginal zone lymphoma), MGH (Massachusetts General Hospital), MSKCC (Memorial Sloan Kettering Cancer Center), SFVAHCS (San Francisco Veterans Administration Health Care System), UCSF (University of California San Francisco), UVA (University of Virginia).

**Table 2:** Pathologic characteristics and FISH results of all 222 specimens compared across different biopsy types (fine needle aspiration biopsy, core needle biopsy, and excisional biopsy) with p values.

	Fine needle aspiration biopsy	Core needle biopsy	Excisional biopsy	P value
Number	46	112	64	
Age (mean (SD))	64.47 (15.70)	65.00 (16.66)	60.38 (15.81)	0.178
Gender (%)				0.209
Female	19 (41.3)	42 (37.5)	31 (48.4)	
Male	27 (58.7)	70 (62.5)	33 (51.6)	
Data Access Group (%)				<0.001
MGH	2 (4.3)	19 (17.0)	6 (9.4)	
MSKCC	4 (8.7)	12 (10.7)	0 (0.0)	
SFVAHCS	6 (13.0)	6 (5.4)	1 (1.6)	
Stanford	20 (43.5)	66 (58.9)	54 (84.4)	
UCSF	14 (30.4)	7 (6.2)	3 (4.7)	
UVA	0 (0.0)	2 (1.8)	0 (0.0)	
Indication (%)				<0.001
Additional diagnostic tissue	1 (2.2)	15 (13.4)	27 (42.2)	
Additional tissue for ancillary studies	0 (0.0)	1 (0.9)	2 (3.1)	
Clinical trial	0 (0.0)	1 (0.9)	0 (0.0)	
Initial diagnosis	27 (58.7)	38 (33.9)	11 (17.2)	
Other/unknown	0 (0.0)	2 (1.8)	0 (0.0)	
R/o recurrence	10 (21.7)	19 (17.0)	7 (10.9)	
R/o transformation	7 (15.2)	36 (32.1)	15 (23.4)	
Staging	1 (2.2)	0 (0.0)	2 (3.1)	
Tissue site (%)				0.832
Bone	1 (2.2)	6 (5.4)	2 (3.1)	
Lymph node or related	29 (63.0)	60 (53.6)	41 (64.1)	
Mediastinum	1 (2.2)	6 (5.4)	2 (3.1)	
Organ	7 (15.2)	23 (20.5)	7 (10.9)	
Other or unknown	1 (2.2)	2 (1.8)	2 (3.1)	
Soft tissue	7 (15.2)	15 (13.4)	10 (15.6)	
Final WHO diagnosis (%)				0.206
Burkitt	1 (2.2)	3 (2.7)	1 (1.6)	
DLBCL	31 (67.4)	85 (75.9)	57 (89.1)	
HGBCL	4 (8.7)	3 (2.7)	2 (3.1)	
HGBCL-MYC/BCL2	7 (15.2)	10 (8.9)	2 (3.1)	
Other/unknown	3 (6.5)	8 (7.1)	0 (0.0)	
PMLBCL	0 (0.0)	2 (1.8)	1 (1.6)	
TCHRLBCL	0 (0.0)	1 (0.9)	1 (1.6)	
*MYC* FISH (%)				0.527
negative	33 (71.7)	86 (76.8)	55 (85.9)	
not done	0 (0.0)	0 (0.0)	1 (1.6)	
positive	11 (23.9)	21 (18.8)	7 (10.9)	
unsuccessful	1 (2.2)	3 (2.7)	0 (0.0)	
variant 3’ signal loss	1 (2.2)	1 (0.9)	1 (1.6)	
variant extra 5’ signaling	0 (0.0)	1 (0.9)	0 (0.0)	
*BCL2* FISH (%)				0.274
extra BCL2 signal on add(3)	0 (0.0)	0 (0.0)	1 (1.6)	
negative	28 (60.9)	60 (53.6)	41 (64.1)	
not done	6 (13.0)	14 (12.5)	2 (3.1)	
positive	12 (26.1)	36 (32.1)	20 (31.2)	
unsuccessful	0 (0.0)	2 (1.8)	0 (0.0)	
*BCL6* FISH (%)				0.718
negative	31 (67.4)	70 (63.1)	40 (62.5)	
not done	7 (15.2)	20 (18.0)	9 (14.1)	
positive	8 (17.4)	18 (16.2)	13 (20.3)	
unsuccessful	0 (0.0)	2 (1.8)	0 (0.0)	
variant 5’ signal loss	0 (0.0)	1 (0.9)	2 (3.2)	

CNB (core needle biopsy), DLBCL (diffuse large B-cell lymphoma), EB (excisional biopsy), FNAB (fine needle aspiration biopsy), HGBCL (high-grade B-cell lymphoma, NOS), HGBCL-MYC/BCL2 (high-grade B-cell lymphoma with MYC and BCL2 rearrangements), PMLBCL (primary mediastinal large B-cell lymphoma), TCHRLBCL (T-cell, histiocytic-rich large B-cell lymphoma), MGH (Massachusetts General Hospital), MSKCC (Memorial Sloan Kettering Cancer Center), SFVAHCS (San Francisco Veterans Administration Health Care System), UCSF (University of California San Francisco, UVA (University of Virginia).

All data extracted from pathology reports was entered into REDCap (Research Electronic Data Capture), a secure, encrypted online database. Study data were collected and managed using REDCap electronic data capture tools hosted at Stanford (23, 24). REDCap (Research Electronic Data Capture) is a secure, web-based software platform designed to support data capture for research studies, providing 1) an intuitive interface for validated data capture; 2) audit trails for tracking data manipulation and export procedures; 3) automated export procedures for seamless data downloads to common statistical packages; and 4) procedures for data integration and interoperability with external sources.

At Stanford Cytogenetics Laboratory, FISH was performed on formalin-fixed, paraffin-embedded (FFPE) sections; the area of interest was circled on the corresponding H&E stained slide by the ordering pathologist. ZytoLight (ZytoVision Gmbh, Bremerhaven, Germany) break-apart probe sets were used for *MYC*, *BCL2*, and *BCL6* FISH. The *MYC* probe set was used with orange probe 5’ to *MYC* and green probe 3’ to *MYC* on 8q24.21. *BCL2* (18q21) probe set included 3’*BCL2* in green and 5’*BCL2* in orange. The same probe configuration was used for *BCL6* (3q27): 3’*BCL6* is green, 5’*BCL6* is orange. All FISH results were scored in 100 interphase cells. A rearrangement was reported if 10% or more cells showed a split signal. The results of these FISH tests were compared to existing FISH results on a different specimen when available. Paired specimens were either obtained from the same anatomic site or from contiguous sites e.g. neck lymph node draining the thyroid or CNS lymphoma spreading to the eye.

At the MSKCC, FISH analyses for *MYC, BCL2*, and *BCL6* (Abbott Molecular, Des Plaines, IL) were performed following a standard protocol, as previously described (25). The area of interest was usually circled on the slide, and the cytogenetics lab staff reviewed the slide to confirm a tumor-rich area was tested in all cases. For each probe set, 100 interphase cells were analyzed. A rearrangement was reported if 10% or more cells showed a split signal.

At UCSF, FISH analysis was performed on formalin-fixed paraffin-embedded (FFPE) tissue sections. The area of interest was circled by the ordering pathologist. Abbott Vysis Dual Color Break Apart probe sets (Des Plaines, IL) were used and FISH was set up according to the probe manufacturer’s instructions (https://www.molecular.abbott/int/en/vysis-fish-knowledge-center/fish-on-isolated-nuclei-from-paraffin). FISH signals were imaged and analyzed using MetaSystems software (Medford, MA). For each probe set, 50 interphase cells were analyzed. A *MYC, BCL2*, or *BCL6* rearrangement was reported if 6.0% or more of cells showed split signals.

At SFVAHCS, *MYC, BCL2*, and *BCL6* FISH were performed at Quest Diagnostics in San Juan Capistrano, CA. Because this was an external lab, the area to perform FISH was not specified or circled. FISH was performed using the probes specific for 3q27 (BCL6), 8q24.1 (MYC), 14q32.3 (IGH), and 18q21.1 (BCL2) [Abbott Molecular and SureFISH, Agilent DAKO]. The cutoff values for *BCL6* rearrangement, *MYC* rearrangement, and t(14;18) in the paraffin-embedded tumor tissue are 11%, 15%, and 3%. For each probe set, 100 cells were analyzed.

At MGH, FISH was performed on 5-micron sections of FFPE tissue; an H&E section was reviewed to select regions for hybridization that contained a majority of tumor cells. Break-apart probes (MYC: Vysis LSI MYC Dual Color, Break Apart Rearrangement Probe; BCL2: Leica Kreatech BCL2 Proximal Green [18Q001B495] and BCL2 Distal Red [18Q002B550) probes]; BCL6: Leica Kreatech BCL6 Proximal Green [03Q008B495] and BCL6 Distal Red [03Q007B550] probes) were hybridized and used to calculate the number of cells out of 50 scored containing a rearrangement. A rearrangement was reported if more than 15% of cells showed split signals.

## 3 Results

**Figure 2:**
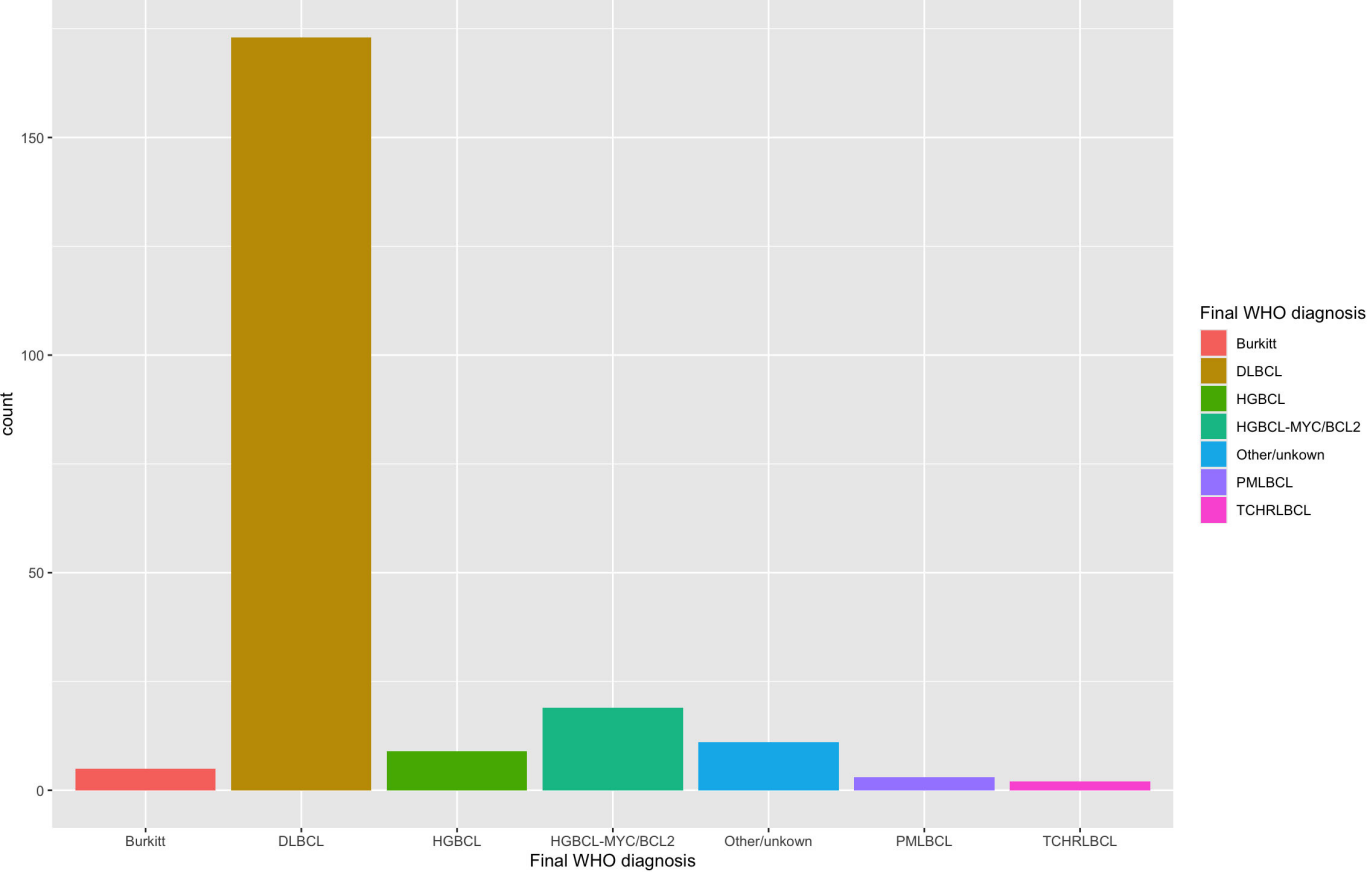
Diagnosis of all large B-cell lymphoma specimens with *MYC, BCL2*, and/or *BCL6* FISH performed. The diagnosis was retrieved retrospectively with FISH results already incorporated. Most cases were diffuse large B-cell lymphoma (DLBCL) (n=173). High-grade B-cell lymphoma with *MYC* and *BCL2* rearrangements (HGBCL-*MYC/BCL2*) (n=19) and high-grade B-cell lymphoma, NOS (HGBCL) (n=9) were smaller subsets. Burkitt lymphoma (n=5), primary mediastinal large B-cell lymphoma (PMLBCL; n=3) and T-cell, histiocyte-rich large B-cell lymphoma (TCHRLBCL; n=2) were rare in this cohort.

A total of 222 specimens from 208 unique patients were identified with a large/high-grade B-cell lymphoma diagnosis and performance of either *MYC*, *BCL2*, or *BCL6* FISH. The clinical characteristics of all patients are described in **Table 1**. The pathologic characteristics of all specimens are compared across different specimen types and corresponding p values in **Table 2**. The final large B-cell lymphoma diagnosis was established after FISH resulted e.g. high-grade B-cell lymphoma with *MYC* and *BCL2* rearrangements or Burkitt lymphoma; the breakdown of different diagnoses can be seen under the header “Final WHO Diagnosis” in **Table 2**. A breakdown of the diagnoses in our cohort is shown in **Figure 2**.

**Figure 3:**
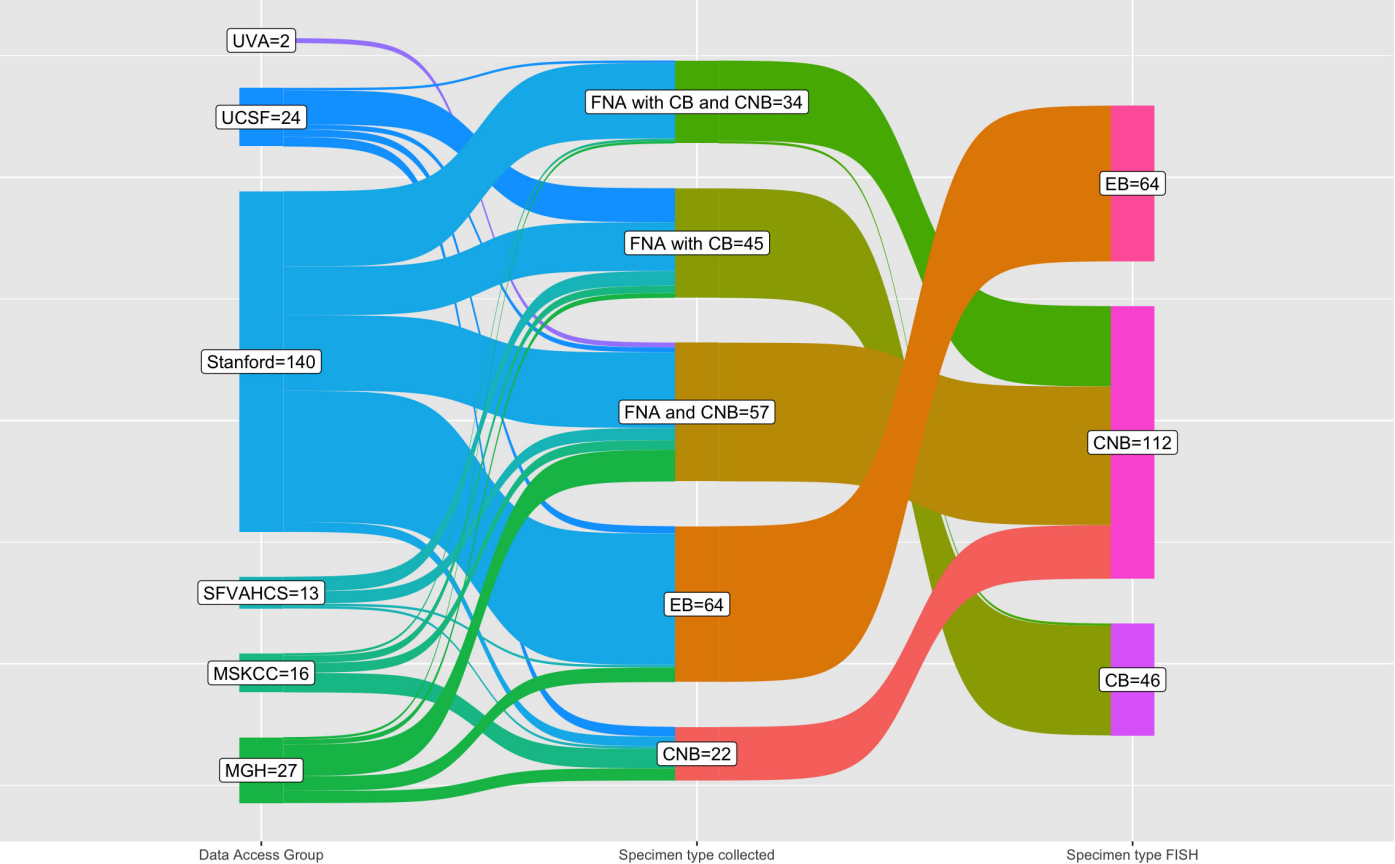
Sankey diagram of specimen type collected and specimen type undergoing FISH testing by institution (data access group). Of all the 222 specimens that underwent FISH testing in this cohort (right column), the majority are core biopsy, followed by excisional biopsy, and cell block. The core biopsy that have FISH performed include, of course, core biopsy only specimens but also FNAB with core biopsy and all FNA with cell block and core biopsy. One FNA with cell block and core biopsy had FISH performed on the cell block due to decalcification of the core biopsy (bone specimen). Abbreviations used: FNA (fine needle aspiration), CB (cell block from FNA), core biopsy (core needle biopsy), EB (excisional biopsy), MGH (Massachusetts General Hospital), MSKCC (Memorial Sloan Kettering Cancer Center), SFVAHCS (San Francisco Veterans Administration Health Care System), UCSF (University of California San Francisco), UVA (University of Virginia).

The breakdown of biopsy specimens by data access group (assigned to academic medical centers as previously described), specimen type collected, and specimen type that had FISH performed is shown by a Sankey diagram in **Figure 3**. Despite the diversity and complexity of some specimen types, for instance, FNAB with cell block and core biopsy with varying degrees of imaging guidance, only three specimen types ultimately had FISH performed: cell block from FNAB, core biopsy, and surgical excisional or incisional biopsy. Most specimens with both a cell block from FNAB and core biopsy had FISH performed on the core biopsy with one specimen having FISH performed on the cell block. Most specimens undergoing FISH were lymph nodes (n=129), followed by non-nodal sites such as solid organs (n=37) or soft tissue (n=32), among others (see **Table 2**). Of the lymph nodes, most were cervical (n=37), retroperitoneal (n=21), inguinal (n=18), or axillary (n=15). All the indications for biopsy are shown in **Table 2**; the four most common indications were initial diagnosis (n=76), obtaining additional diagnostic tissue (n=43), evaluating for recurrence (n=36), and ruling out transformation (n=58).

**Figure 4:**
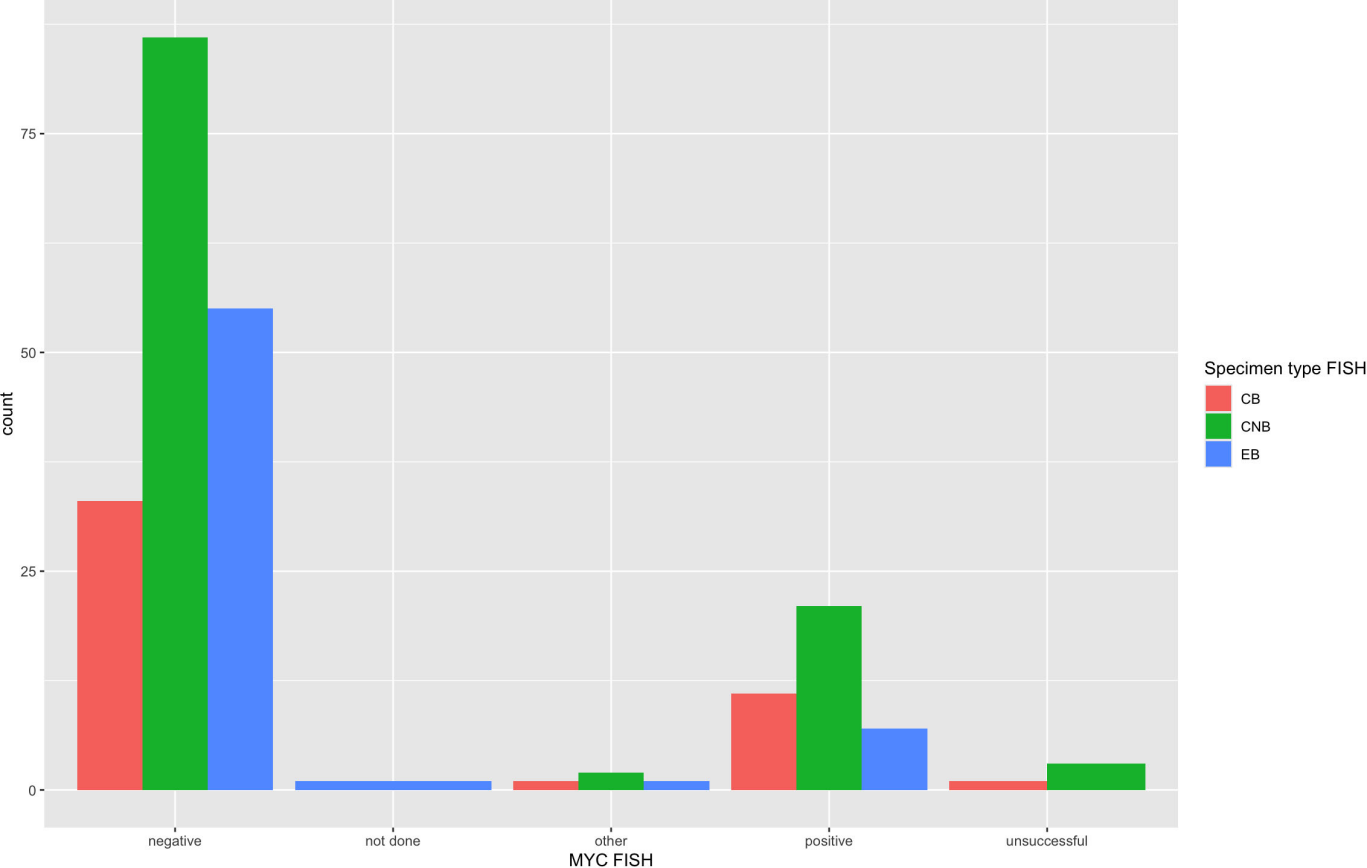
*MYC* FISH results across specimen types. *MYC* FISH results from all 222 specimens are arranged per specimen type that had FISH performed. *MYC* was rearranged in 18% of specimens and showed 4 “other” variant *MYC* FISH results, which included 3 specimens with 3’ signal loss and 1 with extra 5’ signaling. The unsuccessful or hybridization failure rate was overall low at 1.8% and similar across specimen types.

**Figure 5:**
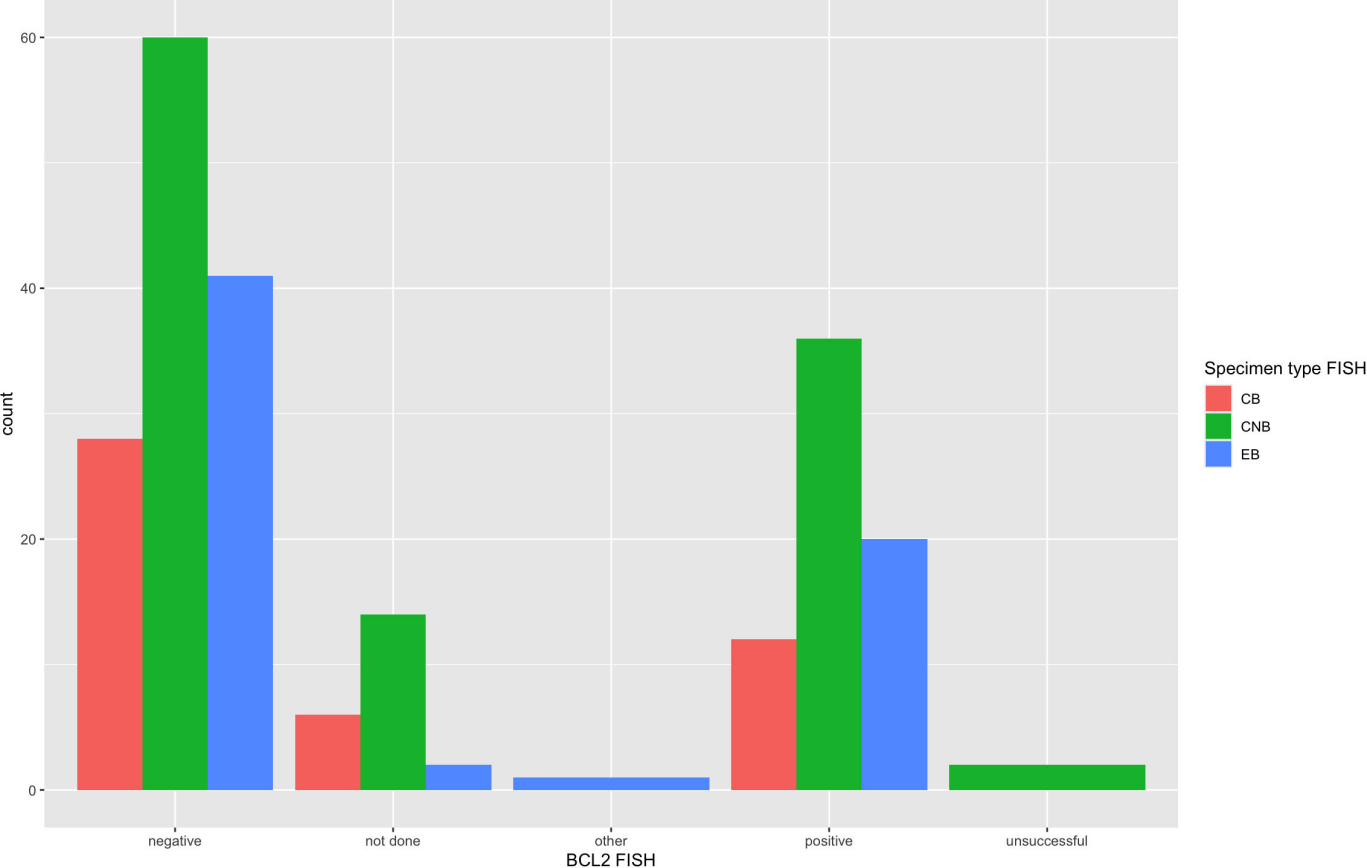
*BCL2* FISH results across specimen types*. BCL2* FISH results from all 222 specimens are arranged per specimen type that had FISH performed. *BCL2* was rearranged in 34% of cases by FISH and showed 1 “other” variant result with 1 extra signal *BCL2* FISH result on an add(3). The unsuccessful or hybridization failure rate was low at 1.0% and similar across specimen types.

FISH detected a *MYC* rearrangement in 39 of 217 specimens successfully tested (18%), no rearrangement in 174 specimens, 4 variant *MYC* FISH results (three with 3’signal loss and one with extra 5’ signaling), and 4 unsuccessful hybridizations (**Figure 4**). One specimen was not evaluated for *MYC* FISH but was evaluated for *BCL2* and *BCL6* FISH; this specimen was included in this study because the patient had a prior biopsy in this cohort that was evaluated for a *MYC* rearrangement, and the additional biopsy was to obtain more tissue for ancillary testing. FISH detected *BCL2* rearrangements in 68 of 198 specimens successfully tested (34%), did not show a rearrangement in 129 specimens, 1 extra signal *BCL2* FISH result on an add(3), 2 unsuccessful hybridizations and 22 specimens without any *BCL2* FISH performed (**Figure 5**). *BCL2* rearrangements were detected in 20 of 37 *MYC* rearranged cases (54%), and these “double-hit” lymphomas are described in the next paragraph. FISH detected *BCL6* rearrangements in 39 of 183 specimens successfully tested (21%), did not show a rearrangement in 141 specimens, 3 specimens with variant 5’ *BCL6* signal loss patterns, 2 unsuccessful hybridizations, and 36 specimens without any *BCL6* FISH performed. The hybridization failure rate was low for *MYC* (4 of 221 or 1.8%), *BCL2* (2 of 200 or 1.0%), and *BCL6* (2 of 185 or 1.1%) probes. Of the 8 unsuccessful hybridizations, 5 (63%) showed tissue limitations such as crush artifact, fibrosis, necrosis, and paucicellularity. FISH results across different biopsy types are shown in **Table 2**.

Concurrent *MYC* and *BCL2* rearrangements were detected in 20 of the 197 specimens (10%) that had FISH successfully performed at both *MYC* and *BCL2* loci; for 19 of these 20 specimens, this result established the diagnosis as high-grade B-cell lymphoma with *MYC* and *BCL2* rearrangements (high-grade B-cell lymphoma-*MYC/BCL2*). One case was indeterminate due to the morphologic differential diagnosis of high-grade B-cell lymphoma-*MYC/BCL2* versus follicular lymphoma with *MYC* and *BCL2* rearrangements, which is not regarded as equivalent to “double-hit” lymphoma according to WHO5/ICC. Of note, 4 additional cases (4 of 197 or 2%) had variant *MYC* FISH results, including 3 with 3’ signal loss and 1 with extra 5’ signaling. All four of these cases with variant *MYC* FISH results also had *BCL2* rearrangements, raising the possibility of high-grade B-cell lymphoma with *MYC* and *BCL2* rearrangements, without a definitive diagnosis. Of the “double-hit” lymphoma cases, 7 had FISH performed on FNA cell blocks, 10 had FISH on core biopsies, and 2 had FISH on the excisional biopsy. The clinical indications for these biopsy specimens were as follows: 7 for ruling out transformation, 7 for initial diagnosis, 4 for ruling out recurrence, and 1 for additional diagnostic tissue. Of note, one of the 19 “double-hit” lymphoma cases showed rearrangements with all three FISH probes *MYC*, *BCL2*, and *BCL6*, which falls in the same diagnostic category as cases with *MYC* and *BCL2* rearrangements only but has been called “triple-hit” lymphoma in the literature. Three specimens of 182 (1.6%) showed *MYC* and *BCL6* rearrangements, which according to the ICC, is diagnostic of the provisional entity high-grade B-cell lymphoma with *MYC* and *BCL6* rearrangements. The WHO5 classification does not recognize this provisional entity but addresses the need for additional data in this patient group.

Of the 222 specimens with FISH performed, 16 paired cytology and surgical specimens were identified from 8 different patients (each patient had exactly one set of paired specimens). An additional 9 patients with paired cytology and surgical biopsy specimens were identified at one institution (Stanford) that had FISH performed on only one set of the pair; completion FISH was performed at Stanford on a research basis on the specimen missing FISH to enlarge the paired specimen cohort. In total, 34 paired specimens were analyzed from 17 patients (**Table 3**). The 17 cytology cases included 13 FNAB with cell block and 4 FNAB with cell block and core biopsy (listed as core biopsy in **Table 3**, because FISH was performed on the core biopsy). The 17 surgical cases were 10 excisional biopsies, 6 core biopsies, and 1 small bowel resection. Of the 102 possible FISH tests (3 different FISH loci across 34 paired specimens), *MYC*, *BCL2*, or *BCL6* FISH was performed in 100 instances (98%). Patient 13 only had *BCL2* FISH performed in both specimens and thus *MYC* and *BCL6* FISH results were not comparable (**Table 3**). Out of the 49 comparisons drawn between these 98 paired FISH tests, 2 comparisons (4%) were discrepant: both showed a *BCL6* rearrangement in the FNA-cell block specimen but not in the paired excisional biopsy from patient 3 or core biopsy from patient 10 (see **Table 3**). No *MYC* or *BCL2* rearrangements were present in any of the discrepant samples. The date of specimens was closely matched for the discrepant pairs, including differences of 5 days and 10 days between the acquisition of the cytology specimen and the surgical specimen. Both patients had the same lymph node sampled by FNA and excisional biopsy or core biopsy. For patient 10, necrosis was noted in the core biopsy with the negative *BCL6* FISH.

**Table 3:** FISH results from 17 paired FNAB or core biopsy and surgical samples are displayed.

Patient	Biopsy 1					Biopsy 2				
	site	Biopsy type	*MYC*	*BCL2*	*BCL6*	site	Biopsy type	*MYC*	*BCL2*	*BCL6*
1	Left axillary	FNA-CB	1	0	0	Lymph nodes (Floor of mouth)	EB	1	0	0
2	Lymph node, left supraclavicular	FNA-CB	0	1	0	Left supraclavicular mass	EB	0	1	0
3	Lymph node, left neck	FNA-CB	0	0	1	Left neck node	EB	0	0	0
4	Lymph node, left neck level 2	FNA-CB	0	0	0	Neck level 2 lymph nodes	EB	0	0	0
5	Right cervical level 2 lymph node	FNA-CB	0	0	0	Lymph node, right neck	EB	0	0	0
6	Left neck lymph node	FNA-CB	0	0	0	Lymph node, left neck	EB	0	0	0
7	Left thyroid	FNA-CB	1	0	1	Left level 5 lymph nodes	EB	1	0	1
8	Lymph node, axillary	FNA-CB	1	0	0	Lymph node, cervical	EB	1	0	0
9	Vitreous	FNA-CB	0	0	0	Brain	EB	0	0	0
10	Lymph node, axillary	FNA-CB	0	0	1	Lymph node, axillary	CNB	0	0	0
11	Lymph node, inguinal	FNA-CB	0	0	0	Lymph node, inguinal	CNB	0	0	0
12	Bone	FNA-CB	1	1	0	Bone	CNB	1	1	0
13	Soft tissue, other or unknown	FNA-CB	0	1	0	Chest wall	CNB	ND	1	ND
14	Right lower quadrant abdomen	CNB	1	0	0	Small Bowel	Resection	1	0	0
15	Pelvis	CNB	0	0	0	Iliac lymph node biopsy	EB	0	0	0
16	Mesentery	CNB	0	1	0	Lymph node, left mesenteric	CNB	0	1	0
17	Left abdominal wall	CNB	0	0	0	Left abdominal mass	CNB	0	0	0

FISH results are reported qualitatively as 0 (negative), 1 (positive), or ND (not done). FISH results are colored green for concordance between small volume biopsy and surgical, red for discordance, and yellow for indeterminate because both specimens were not tested for the same probe. All cases are matched for anatomic site. Two discrepancies were noted between an FNA with cell block (FNA-CB) and surgical case (see BCL6 FISH result for patient 3 and 10); the discrepancy for patient 10 is likely attributable to necrosis noted on the core biopsy specimen causing a false negative FISH result.

FNAB-CB (fine needle aspiration biopsy with cell block), CNB (core needle biopsy), EB (excisional biopsy).

## 4 Discussion

### 4.1 MYC, BCL2, and BCL6 FISH demonstrate highly successful hybridization rates across all specimen types with no statistically significant difference noted between FNAB, core biopsy, and surgical excisions. Tissue limitations may explain the rare failures

The FISH failure rate was low for all three FISH probes: 1.8% for *MYC* (4 of 220), 1.0% for *BCL2* (2 of 208), and 1.1% for *BCL6* (2 of 187). The hybridization rate is essentially the same across small-volume specimens such as FNAB cell block and core biopsy and larger-volume specimens such as an excisional biopsy with a statistically non-significant p-value across all three specimen types (see **Table 2**). **Figures 4**, **5** also demonstrate the breakdown of *MYC* and *BCL2* FISH results across various specimen types and graphically show that hybridization failure rates are low and similar across specimen types. The 8 FISH probe failures were from 6 specimens (one case had failure at all three FISH probes). These 6 specimens consisted of 5 core biopsies (all of which also had FNABs and one of which also had a cell block) and 1 cell block from FNAB. Five of the 6 specimens with failed FISH attempts were noted to have tissue limitations such as crush artifact, fibrosis, necrosis, and paucicellularity, any or all of which may partially explain why these cases had probe hybridization failure.

### 4.2 High-grade B-cell lymphoma with MYC and BCL2 rearrangements was identified across all specimen types

*MYC* FISH detected a rearrangement in 18% of specimens, which is similar to prior series (1, 8, 9, 26, 27). High-grade B-cell lymphoma with *MYC* and *BCL2* rearrangements comprised 9% of all large B-cell lymphoma specimens in our cohort, typical of the 8–10% rate reported in the literature (10, 28). Identification of this subset is critical due to the more aggressive clinical behavior that prompts more aggressive therapy. **Figure 1** illustrates images from a case that was called diffuse large B-cell lymphoma at diagnosis but was later refined to HGBL-*MYC/BCL2* based on FISH results; the images demonstrate three preparations routinely made for FNAB samples of lymph nodes in many cytopathology practices—H&E-stained section of cell block, Pap-stained smear slide (alcohol fixed), and May-Grünwald Giemsa-stained slide (air dried). *MYC* break-apart FISH was performed on the cell block of this FNAB specimen and is depicted. Morphology and even immunohistochemistry are poor predictors of high-grade B-cell lymphoma-*MYC/BCL2*, and FISH or other comparable fusion detection assay such as targeted or whole genome next generation sequencing (12, 29, 30), RNA based sequencing (31), or integrated DNA/RNA sequencing (32) must be performed to identify this important subset of large B-cell lymphoma (28).

Rare variant signal patterns were found with both *MYC* and *BCL2* FISH probes, including signal loss and gain. Our cohort includes 3 patients with variant *3’MYC* loss and 1 patient with *5’MYC* poly-signaling; all the patients in our cohort with variant *MYC* signaling had concurrent *BCL2* rearrangements, raising the possibility of whether these were “double-hit” lymphoma. In general, a scarcity of literature and clinical outcomes about these rare cases exists (33). Copy number variations of *MYC* and *BCL2* have been previously shown to have different biology than structural rearrangements of both genes (13). Another study of variant *MYC* translocations in aggressive B-cell lymphomas found patients with *5’MYC* gain were more refractory to chemotherapy or had an early relapse with a median event-free survival of only 6 months compared to patients with *3’MYC* deletion who often responded to chemotherapy and had an event-free survival of 24 months (34). This study suggested based on survival data and the presence of *IGH/MYC* fusions or other *IGK, IGL* rearrangements in a subset that *5’MYC* gain likely represents an unbalanced *MYC* rearrangement whereas the *3’MYC* deletions were likely unrelated to *MYC* rearrangement. Based on this data, our cases with *3’MYC* signal loss should be excluded from the “double-hit” lymphoma category. This data also suggests our *5’MYC* gain case could be included in “double-hit” lymphoma, but given the overall lack of data and consensus in the literature at this point, the 5’*MYC* gain case was not included in the “double-hit” lymphoma category for our study.

High-grade B-cell lymphoma with *MYC* and *BCL6* rearrangements was much less common in our cohort with 3 cases out of 182 specimens rearranged at both loci (1.6%). As previously mentioned, the significance of these cases is currently controversial. Some studies have not shown distinct biology for these cases (1, 2), but other studies have found an association with a poor outcome (3, 8–11). An additional 3 specimens show variant *BCL6* rearrangements, all 5’BCL6 signal loss, but these specimens were *MYC* FISH negative. Additional studies are needed to further clarify the biology, clinical outcomes, and significance of these cases.

FISH may fail to identify a subset of diffuse large B-cell lymphoma and high-grade B-cell lymphoma that have inferior clinical outcomes. Gene expression profiling of germinal center B-cell diffuse large B-cell lymphoma can identify a double hit-like signature and inferior outcomes, but only half of these cases have structural rearrangements that can be detected by routine *MYC* and *BCL2* break-apart FISH (5). Whole genome sequencing of these cases revealed cryptic *MYC* and *BCL2* rearrangements, copy number gains and amplifications of *MYC* and *MIR17HG*, and focal deletions of the *PVT1* promoter (5). While other technologies in the future may more effectively detect biologically equivalent “double-hit” lymphoma, FISH currently remains the current clinical gold standard for detecting “double-hit” lymphoma.

### 4.3 Paired specimens demonstrate 96% concordance with *MYC, BCL2, and BCL6* FISH results across FNABs, core biopsies, and excisional biopsies

When matched for anatomic site and tissue limitations, paired cytology and surgical specimens in our study showed 96% concordance for FISH results (**Table 3**). Two FISH discrepancies were found and both showed the following pattern: *BCL6* rearrangement was detected in the FNAB while no *BCL6* rearrangement was detected in the paired surgical specimen. Necrosis was noted in the core biopsy from patient 10, and this core biopsy yielded a negative BCL6 result, suggesting that this may be a false negative result. Because no *MYC* rearrangements were present in any of the discrepant samples, the diagnosis would not have changed whether a *BCL6* rearrangement was or was not present.

Overall, this paired data suggests that FNAB cell block is a reasonable alternative to core biopsy or even excisional biopsy for diffuse large B-cell lymphoma and high-grade B-cell lymphoma FISH testing. No *MYC* or *BCL2* FISH discrepancies were found in any pair and, therefore, assessment for “double-hit” lymphoma would not have changed. The only discrepancies between paired samples were at the *BCL6* FISH locus; one of these discrepancies ultimately was attributed to a confounding variable described above.

A major limitation of this study is the heterogeneity of this retrospective and multi-institutional data set, which may limit applicability to some cytogenetic labs and pathology practice settings. Each institution used a different FISH lab with different probe sets and acquisition systems, different split signal thresholds for establishing the presence of a rearrangement, different numbers of interphase cells analyzed, and so on; these differences are reflected in the methods section. The biopsy specimens also have variable indications, which range from initial diagnosis to recurrence or transformation in the post-therapy setting. The data was collected from academic medical centers with highly specialized proceduralists and pathologists subspecializing in cytopathology and hematopathology, which may limit applicability to the community practice setting.

## 5 Conclusions

*MYC*, *BCL2*, and *BCL6* FISH have highly successful hybridization rates that are similar across different specimen types in this cohort, including FNAB, core biopsy, and excisional biopsy. High-grade B-cell lymphoma with *MYC* and *BCL2* rearrangements was detected in 9% of all large B-cell lymphoma specimens, including one case with rearrangements at all three loci *MYC, BCL2*, and *BCL6*; *MYC* and *BCL6* rearrangements were found in 1.6% of specimens. No significant difference was found across biopsy types. Paired cytology and surgical specimens demonstrated 96% concordance at all three *MYC, BCL2*, and *BCL6* FISH loci. FNAB with cell block is an equally effective alternative to core biopsy and excisional biopsy for assessment of *MYC, BCL2, and BCL6* FISH, which is required for identification of the clinically aggressive subset of large B-cell lymphomas that carry both *MYC* and *BCL2* rearrangements.

## Data availability statement

The raw data supporting the conclusions of this article will be made available by the authors, without undue reservation.

## Ethics statement

The studies involving humans were approved by Stanford University IRB protocol 38992. The studies were conducted in accordance with the local legislation and institutional requirements. Written informed consent for participation was not required from the participants or the participants’ legal guardians/next of kin in accordance with the national legislation and institutional requirements.

## Author contributions

JM: Writing – review & editing, Writing – original draft, Visualization, Supervision, Software, Project administration, Methodology, Investigation, Formal analysis, Data curation, Conceptualization. UA: Writing – review & editing, Writing – original draft, Methodology, Data curation. CB: Writing – review & editing, Writing – original draft, Methodology, Data curation. SC: Data curation, Methodology, Writing – original draft, Writing – review & editing. SG: Data curation, Project administration, Writing – original draft, Writing – review & editing. RH: Conceptualization, Data curation, Supervision, Writing – original draft, Writing – review & editing. CK: Writing – review & editing, Writing – original draft, Supervision, Conceptualization. OL: Data curation, Supervision, Writing – original draft, Writing – review & editing, Conceptualization. SL: Conceptualization, Data curation, Supervision, Writing – original draft, Writing – review & editing. AL: Writing – review & editing, Writing – original draft, Supervision, Data curation, Conceptualization. JM: Formal analysis, Methodology, Software, Visualization, Writing – original draft, Writing – review & editing. YN: Supervision, Writing – original draft, Writing – review & editing. RR: Writing – original draft, Writing – review & editing. ES: Writing – review & editing, Writing – original draft, Methodology, Investigation. JY: Data curation, Writing – original draft, Writing – review & editing. SZ: Data curation, Writing – original draft, Writing – review & editing. DG: Writing – review & editing, Writing – original draft, Visualization, Validation, Supervision, Software, Resources, Project administration, Methodology, Investigation, Funding acquisition, Formal analysis, Data curation, Conceptualization.
